# Behandlung von Suchterkrankungen im Strafvollzug

**DOI:** 10.1007/s00103-025-04149-8

**Published:** 2025-10-28

**Authors:** Michael Soyka

**Affiliations:** Psychiatrische Klinik, Universität München, Nussbaumstr. 7, 80336 München, Deutschland

**Keywords:** Opioide, Opiatkonsumstörung, Buprenorphin, Methadon, Gefängnis, Behandlung, Opioids, Opioid use disorder, Buprenorphine, Methadone, Prison, Treatment

## Abstract

Die Assoziation von Substanzkonsumstörungen mit dissozialem Verhalten und Delinquenz ist empirisch gesichert. Alkoholkonsumstörungen und Drogenabhängigkeit sind bei Strafgefangenen häufig und viele suchtkranke Straftäter müssen im Gefängnis betreut werden. Zu den medizinischen Aufgaben gehört hierbei eine adäquate Diagnostik von Suchtmittelkonsum und Folgestörungen, die Behandlung von Entzugssymptomen und Vermittlung oft externer Beratungsangebote. Im Gefängnis gibt es jedoch keine Spezialstationen und wenige spezielle Therapieangebote.

Ein Schwerpunkt der Behandlung von Substanzkonsumstörungen im Gefängnis stellt die Opiatabhängigkeit dar. Die Substitutionsbehandlung ist hierfür die anerkannte First-Line-Therapie, ihre Wirksamkeit ist auch bei Inhaftierten gut belegt. Das Angebot mit oralen oder Depotpräparaten für opiatabhängige Strafgefangene sollte weiter ausgebaut werden. Stark defizitär ist bislang das Behandlungsangebot für Alkoholabhängige oder andere Suchtkranke im Gefängnis, das meist durch externe Suchtberatungsstellen wahrgenommen wird. Im Beitrag werden Grundzüge der medizinisch-therapeutischen Versorgung Inhaftierter mit Substanzkonsumstörungen dargestellt.

## Abstract (en)

Substance use disorders are often associated with antisocial behaviour and criminality. Consequently, alcohol and drug use disorders are frequently noticed in prisoners and many of them require treatment in prison. Adequate management and therapy of affected individuals include diagnosis of substance use and comorbid disorders, management of withdrawal symptoms and implementation of follow-up therapy. However, there are no established specific treatment interventions in prison.

For opioid use disorders, opioid maintenance treatment is the established first-line treatment, and its effectiveness has also been documented in correctional settings. The range of oral and depot preparations available should be further expanded. To date, there has been a significant lack of treatment options for convicted individuals with alcohol use disorder. Some diagnostic and therapeutic advice is provided by external counsellors and social workers. Key principles of treatment in substance use disorders in prisoners are reviewed in this article.

## Einführung

Substanzkonsumstörungen (Suchterkrankungen) spielen für die Gesundheit, Mortalität, aber auch die Kriminalitätsbelastung in der Bevölkerung eine überragende Rolle [1, 2]. Abgesehen vom Nikotinkonsum/Tabakrauchen sind Alkoholkonsumstörungen quantitativ in der Allgemeinbevölkerung am häufigsten. Ihre Rolle für Delinquenz und Kriminalitätsbelastung ist evident [3–6]. Das Thema ist von der deutschsprachigen Forschung mit wenigen Ausnahmen eher vernachlässigt worden. Immerhin hat die Fachgesellschaft Deutsche Gesellschaft für Psychiatrie, Psychotherapie und Nervenheilkunde (DGPPN) vor einigen Jahren ein eigenes Referat „Gefängnispsychiatrie“ gegründet. Relevante Publikationen oder Studien aus diesem Bereich liegen aber noch nicht vor. International hat das Thema Drogen und Gefängnis zum Beispiel die European Union Drugs Agency (Drogenagentur der Europäischen Union, EUDA, vormals EMCDDA) beschäftigt und zu einer Reihe von EU-Monografien geführt [7].

In diesem Beitrag werden zunächst die diagnostischen Kriterien und die Epidemiologie von Suchterkrankungen in der Allgemeinbevölkerung beschrieben. Anschließend werden Suchterkrankungen bei Straftätern in Deutschland thematisiert und mögliche Therapieoptionen im Strafvollzug erläutert.

### Diagnostische Kriterien für Suchterkrankungen

In der internationalen statistischen Klassifikation für Krankheiten und verwandte Gesundheitsprobleme ICD-10 werden im Kapitel F1 „Psychische und Verhaltensstörungen durch psychotrope Substanzen“ die diagnostischen Kriterien für Intoxikation, schädlichen Gebrauch und Abhängigkeit von Alkohol/Drogen dargestellt. Die in Kürze einzuführende ICD-11 wird an der dichotomen Unterscheidung von schädlichem Gebrauch und Abhängigkeit festhalten [2]. Ein bestimmter Schwellenwert, ab dem ein Konsummuster oder das Trinkverhalten als pathologisch anzusehen ist, wird nicht definiert. Für die Diagnose „schädlicher Gebrauch“ wird explizit eine tatsächliche Schädigung der psychischen und physischen Gesundheit durch Alkohol/Drogen gefordert; soziale Folgeschäden oder eine Intoxikation sind in ICD-10 für die Diagnosestellung nicht ausreichend.

Die ICD-10 folgt einem biopsychosozialen Krankheitskonzept, d. h., die einzelnen Folgeschäden, die sich durch einen Substanzkonsum ergeben, können sich auf der psychischen, körperlichen oder auch sozialen Ebene zeigen. Eine Abhängigkeit liegt vor, wenn in den letzten 12 Monaten 3 oder mehr der folgenden 6 Kriterien erfüllt worden sind:

1. ein starker Wunsch oder eine Art Zwang, Alkohol/Drogen zu konsumieren,
2. verminderte Kontrollfähigkeit bezüglich des Beginns, der Beendigung und der Menge des Konsums,
3. ein körperliches Entzugssyndrom bei Beendigung oder Reduktion des Konsums,
4. erhöhte Toleranz in Bezug auf die Substanzwirkung,
5. fortschreitende Vernachlässigung anderer Vergnügen oder Interessen zugunsten des Substanzkonsums,
6. anhaltender Konsum trotz Nachweis eindeutiger sozialer Schädigungen.

Im psychosozialen Bereich kommen zum Beispiel Arbeitslosigkeit, Scheidung, Führerscheinverlust, aber auch Aggression und Suizidgefährdung hinzu.

Die neue ICD-11 ist auf Deutsch noch nicht verfügbar [2, 9]. Bislang liegt nur eine vorläufige deutsche Übersetzung vor.^1^ Die einzelnen Störungsbilder werden in der neuen ICD-11 anders hierarchisiert und kodiert.

Neu eingeführt wird die Kategorie „riskanter Konsum“ (psychoaktive Substanzen: ICD-11-Code: QE 11; andere spezifische Substanzen: QE 1Y) im Kapitel „Probleme in Verbindung mit dem Gesundheitsverhalten“ (ICD-11 24). Riskanter Konsum wird durch Menge und Konsumfrequenz, Konsumart und den Kontext des Konsums definiert, wenn sich daraus schädliche physische oder psychische Konsequenzen ergeben können.

Ein schädlicher Gebrauch (Codierung: 86c 3x.1) liegt nach ICD-11 vor, wenn über mindestens einen Monat kontinuierlich oder innerhalb eines Jahres wiederholt ein Konsummuster besteht, das zu einer physischen oder psychischen Schädigung infolge einer Intoxikation oder direkter oder indirekter toxischer Effekte bei der betroffenen Person oder anderen Personen im Umfeld geführt hat.

Für die Diagnose einer Abhängigkeit nach ICD-11 (6c 4x.2) müssen von 3 definierten Merkmalspaaren, die je 2 definierte Items enthalten, mindestens 2 erfüllt sein, innerhalb der Merkmalspaare jeweils ein Item (Zeitraum von mindestens 12 Monaten oder von mindestens einem Monat, über den die Merkmale kontinuierlich (täglich oder fast täglich) vorgelegen haben):

1. beeinträchtigte Kontrolle über den Substanzkonsum – bezogen auf Beginn, Menge und Umstände oder Ende des Konsums,
2. physiologische Merkmale manifestieren sich als: (i) Toleranz, (ii) Entzugserscheinungen nach Konsumstopp oder -reduktion oder (iii) wiederholter Konsum der Substanz, um Entzugserscheinungen zu mindern oder zu verhindern,
3. Substanzkonsum wird fortschreitend zur Priorität im Leben, sodass die Substanz Vorrang über andere Interessen, Vergnügungen, alltägliche Aktivitäten, Verpflichtungen oder die Gesundheits- oder persönliche Pflege erhält.

### Epidemiologie

Alkoholkonsumstörungen sind häufig, mindestens 3 % der erwachsenen Bevölkerung in Deutschland sind alkoholabhängig, wobei die Trinkmenge meist sehr stark erhöht ist [8]. Die meisten Patienten mit Alkoholabhängigkeit sind selten oder gar nicht in Behandlung, nur etwa 10 % der Betroffenen werden fachgerecht behandelt. Hochrechnungen für Deutschland ergaben, dass von den abhängigen Alkoholkonsumenten lediglich zwischen 10,5 % und 22,5 % irgendeine Therapie in Anspruch genommen haben [8].

Der Pro-Kopf-Konsum für die Bevölkerung beträgt rund 10 l Alkohol pro Jahr mit geringem Rückgang in den letzten Jahren [2].

Die 12-Monats-Prävalenz von Alkoholmissbrauch und Abhängigkeit nach den damaligen Kriterien des Klassifikationssystems für psychische Störungen der American Psychiatric Association (DSM IV; [2, 9]) in der Allgemeinbevölkerung lag bei 3,0 % für Abhängigkeit und 1,8 % für Missbrauch. Männer waren mit 4,4 % bzw. 3,1 % wesentlich häufiger betroffen als Frauen mit 1,6 % bzw. 0,4 %. Dazu kommt eine große Gruppe von Menschen, die riskanten Alkoholkonsum betreiben, also zu viel trinken, ohne Folgeschäden zu haben oder abhängig zu sein. Hochgerechnet auf die Bevölkerung Deutschlands weisen etwa 7,9 % einen riskanten (potenziell gesundheitsschädlichen) Konsum auf [10]. Ein problematischer Alkoholkonsum lag nach dem Epidemiologischen Suchtsurvey 2021 bei 17,6 % der Bevölkerung vor [11].

Beim Konsum illegaler Substanzen (einschließlich Cannabis) haben sich in den letzten Jahren einige Veränderungen ergeben. Cannabiskonsum (und -besitz), mittlerweile weitgehend halb legalisiert, hat eine Lebenszeitprävalenz von 27,9 % bei den 15- bis 24-Jährigen in Europa. Die Prävalenz für Opiatabhängigkeit liegt bei etwa 0,4 %. Nach epidemiologischen Schätzungen konsumieren zahlreiche Menschen auch psychotrope Substanzen. Etwa 1,1 Mio. Deutsche gebrauchen Opioid-Analgetika und 2,8 Mio. Schlaf- oder Beruhigungsmittel [2].

Bei den sogenannten Drogentoten spielen Opioide nach wie vor die größte Rolle aufgrund ihres atemdepressiven Effekts. Nach dem europäischen Drogenbericht (EMCDDA 2022 [7]) wurden bei 74 % der drogenassoziierten Todesfälle Opioide nachgewiesen.

Die Zahl der Kokain- und Crackkonsumenten wird in Deutschland etwa auf 818.000 geschätzt [10], der von Amphetaminkonsumenten auf 716.000, wobei der Konsum von Kokain und auch anderen Psychostimulanzien im Gegensatz zu Opioiden eher anzusteigen scheint [11].

Ein großes Problem bei den drogeninduzierten Störungen stellen die sogenannten neuen psychoaktiven Substanzen (NPS) dar, auch als „legal highs“ bezeichnet [2]. Es gibt mittlerweile eine Vielzahl von synthetischen Cannabinoiden, aber auch Halluzinogene oder andere Substanzen, wie z. B. nicht zugelassene Benzodiazepine, die den Markt überschwemmen und überwiegend über das Internet bezogen werden können. Sie spielen auch in Strafanstalten eine große Rolle [12].

### Sucht und Delinquenz

Alkohol spielt bei Straftaten, insbesondere Körperverletzung und Tötungsdelikten, eine enorme Rolle [4–6]. Die Alkoholbelastung und der Prozentanteil alkoholisierter Tatverdächtiger ist aus der jährlich erscheinenden polizeilichen Kriminalstatistik zu ersehen. Außerdem spielen Alkohol und andere Rauschdrogen eine große Rolle vor allem bei schweren Autounfällen [13].

Für Gewaltkriminalität liegt die Rate der alkoholisierten Täter bei etwa 21 %, für Totschlag bei rund 25,5 %, für Sachbeschädigung bei 18,7 %. Besonders hoch ist, wenig überraschend, die Rate der Alkoholisierten bei Widerstand gegen die Staatsgewalt (rund 49,9 %). Bei Straftaten insgesamt beträgt der Anteil alkoholisierter Tatverdächtiger 9,6 %. Alkohol wirkt enthemmend, zum Teil aggressionsfördernd, vor allem bei schneller Anflutung.

Es ist empirisch gesichert, dass auch andere Rauschdrogen für die Kriminalität relevant sind – zum einen wegen drogeninduzierter Intoxikationen, zum anderen auch indirekt im Rahmen der sogenannten Beschaffungskriminalität als Tatmotiv. Die meisten Drogenabhängigen können ihren Drogenkonsum nur durch weitere Straftaten (Drogenhandel, Einbrüche etc.) aufrechterhalten. Die Drogen selber führen, je nach eingenommener Substanz, zu unterschiedlichen Auffälligkeiten wie (Übersicht in [6]):

- Beeinträchtigung von Selbstbeherrschung,
- Bewusstwerden unterdrückten Ärgers,
- Beeinträchtigung der Urteilsfähigkeit,
- Beeinträchtigung der Impulskontrolle,
- Unruhe,
- Reizbarkeit,
- Affektlabilität,
- verstärktes Misstrauen,
- sehr selten auch paradoxe Reaktionen im Sinne eines pathologischen Rausches (fehlende Korrelation zur aufgenommenen Menge).

Vor diesem Hintergrund überrascht es nicht, dass viele Täter in Haftanstalten alkohol- oder drogenabhängig sind. Laut aktueller Kriminalstatistik 2024 hat die Rauschgiftkriminalität um 34,2 % abgenommen, vor allem aufgrund der neuen Cannabis-Regelung. Kokain spielt dagegen eine zunehmende Rolle.

Das Thema Sucht im Gefängnis hat auch den wissenschaftlichen Dienst des Deutschen Bundestags beschäftigt, der 2022 eine Dokumentation zur Versorgung suchtkranker Personen im Justiz- und Maßregelvollzug herausgegeben hat.^2^

Die wissenschaftliche Evidenz zur Assoziation von Substanzkonsumstörungen und Kriminalität ist eindeutig. Zur Frage Substanzmissbrauch und Delinquenz führten Fazel et al. [4] ein systematisches Review durch; eingeschlossen wurden 24 Studien mit 18.388 Gefangenen. Die Prävalenzraten für Drogenmissbrauch und -abhängigkeit lagen bei 30 % bei Männern und sogar bei 51 % bei Frauen. In Deutschland wurde von Opitz-Welke et al. (2018) berichtet, dass etwa 20 % der Gefängnisinsassen opiatabhängig sind [14].

Es gibt eine jährliche bundeseinheitliche Erhebung zur stoffgebundenen Suchtproblematik bei Gefangenen bzw. zur stoffgebundenen Suchtproblematik in bundesdeutschen Haftanstalten, die 49.915 der 54.550 im Justizvollzug untergebrachten Gefangenen umfasst. Eine Substanzabhängigkeit wurde 2023 bei 25 % der Inhaftierten gesehen (bei Männern 24 %, bei Frauen 29 %), ein Substanzmissbrauch bei 15 % (15 % bei Männern, 11 % bei Frauen).^3^ Die missbrauchten Substanzen waren vielgestaltig, am häufigsten waren Cannabis, Opioide, Alkohol (keineswegs dominierend!) und polytoxikomanes Suchtverhalten (gleichzeitige Abhängigkeit von mehreren Substanzen).

Die Substitutionsquote bei Abhängigkeit von Opioiden und multiplen Substanzen wurde mit 45,8 % (*N* = 2906 von 6350) angegeben.^3^

Zum Stichtag 31.03.2023 befanden sich 6555 Personen wegen Verstößen gegen das Betäubungsmittelgesetz (BtMG) in Haft (14,8 % aller Inhaftierten). Der Anteil der Strafgefangenen mit BtMG-Verstößen war über die letzten Jahre rückläufig.

Der REITOX-Bericht (Deutsche Beobachtungsstelle für Drogen und Drogensucht 2024 [15]) nennt eine Zahl von 25 % der Inhaftierten mit einer Abhängigkeit und 15 % mit schädlichem Gebrauch psychotroper Substanzen, einschließlich Alkohol (ICD-10-Kriterien, siehe [2, 9]).

Auch andere Untersuchungen zeigen eine ähnlich hohe Rate von Opiatabhängigkeit bei Straftätern in Haft [16, 17].

### Rechtliche Rahmenbedingungen

Das deutsche Recht kennt die Möglichkeit, Straftätern, bei denen eine Suchterkrankung für die begangene Straftat relevant war, wegen ihrer Sucht bzw. eines „Hanges“, berauschende Mittel „im Übermaß“ zu konsumieren (gemäß § 64 Strafgesetzbuch, StGB), eine Behandlung im Maßregelvollzug, also in einer Entziehungsanstalt, aufzuerlegen.

Eine aufgehobene oder verminderte Einsichts- oder Steuerungsfähigkeit gemäß §§ 20, 21 StGB muss dabei nicht vorliegen. Zahlenmäßig hat die Unterbringung psychisch kranker Straftäter mit Suchterkrankungen nach § 64 StGB in den letzten Jahren epidemische Ausmaße angenommen, was den Gesetzgeber 2024 zu einer Änderung der gesetzlichen Grundlagen veranlasst hat.

Nach § 64 StGB „neu“ muss nun eine Substanzkonsumstörung diagnostiziert werden, die Suchterkrankung muss für das verurteilte Anlassdelikt eine entscheidende Rolle gespielt haben und es muss durch den Konsum zu Funktionseinschränkungen in mehreren Lebensbereichen gekommen sein. Voraussetzung ist auch eine negative Legalprognose (Risiko für weitere erhebliche Straftaten) bei weiterem Konsum [18].

Auch eine positive Behandlungsprognose wird für die Anwendung des § 64 StGB vorausgesetzt.

Mit anderen Worten: Die rechtlichen Hürden für eine Unterbringung psychisch kranker Straftäter mit Substanzkonsumstörung im Maßregelvollzug haben sich deutlich erhöht. Es ist davon auszugehen, dass entsprechend mehr Probanden (Straftäter, bei denen die Strafe zugunsten einer Behandlung im Maßregelvollzug ausgesetzt wird) mit Suchterkrankung im Gefängnis aufgefangen werden müssen.

Hintergrund war u. a., dass etwa 60 % der nach § 64 StGB untergebrachten Probanden schuldfähig waren und bei vielen der untergebrachten Täter eher ein dissozialer Lebensstil denn eine Substanzkonsumstörung vorlag. Zum 01.10.2023 ist die Novellierung des § 64 StGB in Kraft getreten [19, 20].

Der Wortlaut des neuen Gesetzes § 64 StGB:

„Hat eine Person den Hang, alkoholische Getränke oder andere berauschende Mittel im Übermaß zu sich zu nehmen, und wird sie wegen einer rechtswidrigen Tat, die überwiegend auf ihren Hang zurückgeht, verurteilt oder nur deshalb nicht verurteilt, weil ihre Schuldfähigkeit erwiesen oder nicht auszuschließen ist, so soll das Gericht die Unterbringung in einer Entziehungsanstalt anordnen, wenn die Gefahr besteht, dass sie infolge ihres Hanges erhebliche rechtswidrige Taten begehen wird; der Hang erfordert eine Substanzkonsumstörung, infolge derer eine dauernde und schwerwiegende Beeinträchtigung der Lebensgestaltung, der Gesundheit, der Arbeits- oder der Leistungsfähigkeit eingetreten ist und fortdauert.

Die Anordnung ergeht nur, wenn aufgrund tatsächlicher Anhaltspunkte zu erwarten ist, die Person durch die Behandlung in einer Entziehungsanstalt innerhalb der Frist nach § 67d Abs. 1 Satz 1 oder 3 zu heilen oder über eine erhebliche Zeit vor dem Rückfall in den Hang zu bewahren und von der Begehung erheblicher rechtswidriger Taten abzuhalten, die auf ihren Hang zurückgehen.“

### Therapie von Suchterkrankungen

Für die Behandlung von Alkoholkonsumstörungen, medikamentenbezogenen Störungen und in Kürze auch Opiatabhängigkeit (im Konsultationsverfahren) liegen aktuelle S3-Leitlinien vor (siehe Arbeitsgemeinschaft der Wissenschaftlichen Medizinischen Fachgesellschaften e. V., AWMF). Die relevanten Leitlinien betreffen alkoholbezogene Störungen (AWMF 076-001)^4^ und medikamentenbezogene Störungen (038–025)^5^. Die Leitlinie zu Opiatabhängigkeit ist in der Vernehmlassung und dürfte in der nahen Zukunft ebenfalls verfügbar sein.

Die therapeutischen Möglichkeiten bei Alkoholabhängigkeit sind intensiv erforscht worden. Bei längeren stationären Behandlungen sind Erfolgsraten (dauerhafte Abstinenz) bis etwa 40 % erreichbar. Die Therapieforschung hat dabei gezeigt, dass die Erfolgsraten bei Wiederholungsbehandlungen deutlich geringer ausfallen [2]. Die Erfolgsaussichten hinsichtlich langfristiger Abstinenz sind bei Drogenabhängigkeit deutlich geringer als bei legalen Substanzen, speziell Alkohol [2].

#### Therapie im Maßregelvollzug

Die Effizienz der Therapie von Substanzkonsumstörungen im Maßregelvollzug ist etwas kontrovers, es gibt aber immerhin einige empirische Studien, die eine Wirksamkeit der Behandlung nach § 64 StGB im Hinblick auf Suchtmittelkonsum und Kriminalitätsprognose zeigen [20–22]. Die Abbruchrate im Maßregelvollzug beträgt etwa 50 % [23].

Die Substitution mit Opiaten ist in einigen, aber längst nicht allen Einrichtungen des Maßregelvollzugs möglich [24].

### Behandlung von Substanzkonsumstörungen im Gefängnis

Anders als z. B. bei der Behandlung von Sexualstraftätern gibt es in Gefängnissen keine Spezialstationen für Suchtkranke und wenige spezielle Therapieangebote. Die verschiedenen therapeutischen Interventionen bei Inhaftierten mit Substanzkonsumstörung sind idealtypisch in Tab. 1 zusammengefasst.

**Tab. 1:** Therapeutische Interventionen bei Inhaftierten mit Substanzkonsumstörungen

Zeitpunkt	Intervention
Eingangsuntersuchung	Anamnese
	Somatostatus
	Psychostatus
	Prüfung auf Haftfähigkeit
	Ggf. Medikationsplan
	Behandlung von Entzugssymptomen
	Drogenkontrolle (Urin, Blut)
	Infektionsprophylaxe/-kontrolle (Hepatitis B/C, HIV, Tuberkulose)
	Angebot von Impfmöglichkeiten
	Fortführung/Einleitung einer Substitutionsbehandlung
	Behandlung von Folgestörungen
Klinisches Management während des Vollzugs	Behandlung komorbider Störungen
	Substitutionsbehandlung
	Vermeidung von Überdosierungen
	Drogenkontrolle
	Safer-Use-Beratung
	Vernetzung mit externen Hilfsangeboten (Drogenberatung, Aidshilfe, ggf. Selbsthilfegruppen)
	Sozialdienst, Schuldnerberatung
	Aufnahme in Spezialabteilung, falls vorhanden
	Ggf. Drogennotfalltraining (Naloxon)
Entlassmanagement	Vernetzung mit externer Suchtberatung
	Therapieeinleitung
	Fortführung Substitution
	Hilfeplanung, Sozialdienst

Die medizinische Versorgung der Betroffenen wird durch das örtliche medizinische Personal, gelegentlich auch durch einen hinzugezogenen Psychiater vorgenommen. Regelmäßig werden Inhaftierte auf Wunsch im Gefängnis von Beratungsstellen (externe Suchtberatung) aufgesucht, die über weitergehende Therapieangebote für Suchterkrankungen informieren und ggf. auch Anträge für weitere Therapiemaßnahmen stellen (Kostenübernahme etc.). Die Finanzierung der externen Suchtberatung ist allerdings nicht überall gesichert, aktuell läuft zu diesem Problem eine Petition (www.openpetition.de). Für Jugendliche und junge Erwachsene mit problematischem Cannabiskonsum wurde von 2008 bis 2011 immerhin in Jugendstrafanstalten ein evidenzbasiertes sekundärpräventives Gruppenbehandlungsprogramm überprüft [25]. Einige wenige Haftanstalten haben spezielle Behandlungsabteilungen für Betroffene.

#### Alkohol

Obwohl die Zahl von Strafgefangenen mit Alkoholkonsumstörungen weit größer ist als von denjenigen mit problematischem Konsum illegaler Substanzen wird in Übersichten zu dem Thema fast ausschließlich auf Drogenabhängige Bezug genommen [26, 27].

Für die Behandlung von Alkoholkonsumstörungen in Gefängnissen bestehen keine besonderen Therapieangebote und Alkoholkonsum selber ist, im Gegensatz zu dem weitverbreiteten Konsum sogenannter illegaler Substanzen, im Gefängnis eher selten. Zur Pharmakotherapie der Alkoholabhängigkeit sind nur relativ wenige Substanzen zugelassen, namentlich Acamprosat sowie die Opioidantagonisten Naltrexon und Nalmefen [28]. Diese finden aber in der medizinischen Behandlung Alkoholabhängiger in Gefängnissen praktisch keine Anwendung.

#### Drogenabhängigkeit

Ein gewisser Schwerpunkt der Behandlung von Substanzkonsumstörungen im Gefängnis stellt die Opiatabhängigkeit dar. Opiatkonsum geht mit einer hohen Mortalität einher, erst recht bei Patienten mit fehlender pharmakologischer Toleranz. Ein besonders kritischer Zeitpunkt besteht unmittelbar nach der Entlassung [29]. Bei nichtsubstituierten Inhaftierten fehlt eine Toleranz für die atemdepressive Wirkung von Opiaten. Die Substitutionsbehandlung von Opiatabhängigen ist der unbestrittene Goldstandard in der Therapie [29, 30]. Diese ist im Gefängnis bisher möglich und wurde auch in verschiedenen Gefängnissen, allerdings nicht in allen Bundesländern, praktiziert. Dabei kamen sowohl Methadon als voller μ‑Opiatagonist sowie Buprenorphin als partieller Agonist zur Anwendung. Beide Substanzen sind bei oraler/sublingualer Gabe 24 h wirksam und verhindern Entzugserscheinungen oder Verlangen nach Opiaten.

Methadon ist ein synthetisch hergestelltes Analgetikum und ein Agonist am μ‑Opioidrezeptor. Typische Dosierungen sind 60–120 mg Methadon beziehungsweise 30–60 mg Levomethadon pro Tag. Toleranzentwicklung und auch Euphorie sind unter Methadon häufig, wenngleich nicht so stark ausgeprägt wie bei Heroin. Hauptrisiko sind Überdosierungen mit Atemstillstand.

Buprenorphin ist ebenfalls ein stark wirksames Analgetikum. Buprenorphin ist ein Agonist am μ‑Opioidrezeptor und hat am κ‑Opioidrezeptor eine gemischt agonistisch-antagonistische Wirkung. Übliche Dosierungen liegen zwischen 8 mg und 16 mg (max. 24 mg). Buprenorphin steht als Monosubstanz und als Kombinationsmedikament mit Naloxon im Verhältnis 4:1 zur Verfügung. Eine Sublingualtablette mit 8 mg Buprenorphin enthält 2 mg Naloxon. Naloxon soll den intravenösen Konsum verhindern, da dieser einen Opiatentzug auslösen würde.

Typische und häufige Nebenwirkungen von Methadon sind Benommenheit, Schläfrigkeit, Übelkeit, Erbrechen, Ödeme, auch Harnverhalt und Obstipation. Im EKG können QT-Zeit-Verlängerungen auftreten. Auch Buprenorphin kann ähnliche, opiattypische Nebenwirkungen verursachen. Das Risiko für Sedierung ist im Vergleich zu Methadon geringer.

Ein großes Problem, insbesondere unter den konkreten Bedingungen des Gefängnisses, ist, dass Drogen dort nicht nur als Rauschmittel, sondern auch als Druckmittel und Ware (Weitergabe von Drogen) fungieren. Die Drogenbelastung einiger Strafanstalten ist sehr hoch.

Ein gewisser „Gamechanger“ im Hinblick auf das Risiko der Weitergabe von Substitutionsmitteln stellt die Einführung von Depotpräparaten namentlich von Buprenorphin dar [31]. Hier sind seit einigen Jahren Depotspritzen verfügbar [31], die bei guter Verträglichkeit in der Wirksamkeit mit dem sublingual gegebenen Buprenorphin vergleichbar sind. Durch diese entfällt das Risiko der Weitergabe, ein weiterer Vorteil ist die sehr gute Compliance. Insofern werden diese Substanzen im Gefängnis vermehrt eingesetzt.

Generell gibt es eine breite klinisch-wissenschaftliche Literatur zur Therapie von Opiatabhängigkeit in Gefängnissen, allerdings kaum deutschsprachige Studien. Relevant ist die vom Land Bayern geförderte HOPE-Studie zur Effizienz der Substitutionsbehandlung im Gefängnis [32]. Hierbei handelt es sich um eine nichtrandomisierte Vergleichsuntersuchung von substituierten und nichtsubstituierten opiatabhängigen Strafgefangenen. Im Rahmen der HOPE-Studie wurden 247 Gefangene in Bayrischen Haftanstalten sowie Justizvollzugsbeamte befragt. Da keine randomisierte klinische Untersuchung durchgeführt wurde, kann der Wirknachweis der Opioidsubstitution in Haftbedingungen hier nicht abschließend beurteilt werden [32]. Insgesamt war der Behandlungsprozess mit Drogenersatzstoffen während der Haft aus Sicht der Untersucher wirksam im Sinne einer Reduktion von illegalem Konsum und Verlangen sowie geregelter Anschlussbehandlung. Die Rezidivrate hinsichtlich erneuter Straftaten in der Studie war allerdings niederschmetternd hoch (40 % in 3 Jahren).

Bei Opiatabhängigkeit besteht das Risiko, dass in der Haft, vor allem aber nach der Entlassung, bei den Betroffenen schwere Intoxikationen oder auch Todesfälle bei Drogenrückfall auftreten. Es konnte klinisch in verschiedenen Studien gezeigt werden, dass die Initiierung oder Fortführung einer bereits auswärts begonnenen Substitutionsbehandlung das Risiko der Mortalität deutlich senkt, zum Teil auch das Risiko für Infektionserkrankungen oder andere drogenassoziierte Folgeschäden [33, 34]. Der Effekt auf die Kriminalprognose ist weniger eindeutig. Es gibt einige wenige randomisierte Vergleichsuntersuchungen von substituierten vs. nichtsubstituierten Inhaftierten und eine ganze Reihe longitudinaler Studien [35]. Diesbezüglich liegt eine Reihe von aussagekräftigen systematischen Reviews und Metaanalysen vor [36–40].

Für andere Rauschdrogen stehen keine wirksamen Substitutionsmittel oder Antidote zur Verfügung. Dies betrifft insbesondere Kokain, Psychostimulanzien, aber auch Cannabis oder Sedativa. Ein großes Problem stellt zunehmend auch die Verbreitung von NPS im Gefängnisalltag dar [12]. Die Substanzen sind häufig schwer nachweisbar. Meist handelt es sich um neuere, sehr toxische Cannabinoide oder Halluzinogene. Psychotoxische Wirkungen sind häufig.

#### Gesundheitsangebote und Harm-Reduction-Programme

Der REITOX-Bericht 2024 [15] nennt als drogenbezogene Gesundheitsangebote im Gefängnis die medizinische Begleitung/Versorgung/Entgiftung intoxikierter Inhaftierter, die Substitutionsbehandlung bei Bedarf in Justizvollzugsanstalten, ggf. mit psychosozialer Betreuung und die Vorstellung Substituierter bei einem Substitutionsarzt vor der Haftentlassung. Außerdem werden in vielen deutschen Justizvollzugsanstalten verschiedene Träger der Suchthilfe tätig, namentlich in der Beratung und Begleitung Inhaftierter mit einer Suchtproblematik. Der Bericht nennt Abstinenzkontrollprogramme über Urin- und Speicheltestungen in einigen Haftanstalten, zum Teil werden Aufklärungs- und Präventionsmaßnahmen für Drogengebrauch bei Gefangenen bezüglich des Infektionsschutzes angeboten, zum Teil erfolgen auch Testungen auf drogenbezogene Infektionskrankheiten.

Es gibt eine Reihe von sogenannten Harm-Reduction-Ansätzen, die im Gefängnis kaum implementiert sind [27]. Dazu gehört z. B. die Take-Home-Gabe des rasch wirksamen Opioidantagonisten Naloxon, der das Risiko tödlicher Opiatintoxikationen vermindern soll, aber auch sogenannte Spritzen‑/Nadelaustausch-Programme [41]. Die Take-Home-Gabe von Naloxon, das seit 2018 auch als Naloxon-Nasenspray verfügbar ist, soll es geschulten Menschen im Falle einer Überdosierung ermöglichen, eine Atemlähmung zu verhindern und eine Betreuung bis zum Eintreffen von Notfallhelfern/Rettungsdienst zu gewährleisten. Eine Drogennotfalltraining vor Anwendung wird empfohlen. Die Effizienz ist zwar schwer zu belegen, aber es gibt einige Beobachtungsstudien, die eine reduzierte Mortalität bei Opiatabhängigen durch die Gabe von Naloxon nahelegen [42–44].

In Haftanstalten ist dieser Ansatz bislang kaum implementiert. Entsprechende Maßnahmen sind im wissenschaftlichen, aber auch im politischen Raum durchaus gefordert worden [26, 27]. Kaum praktiziert wird eine Kooperation mit Selbsthilfegruppen [26].

## Fazit

Die Assoziation von Suchtmittelkonsum mit Kriminalität ist empirisch gesichert [45, 46], Substanzkonsumstörungen bei Strafgefangenen sind entsprechend häufig. Die Neuregelung des § 64 StGB (Maßregelvollzug) lässt sogar eher steigende Zahlen erwarten. Das therapeutische Angebot bei Alkoholkonsumstörungen ist sehr begrenzt, der Fokus liegt auf der Behandlung Opiatabhängiger, speziell auf der Substitutionsbehandlung. Andere Therapieansätze sind auszubauen. Besonders heikel ist, wie oft in der psychiatrischen Therapie, die Schnittstellenproblematik, also die optimale Diagnostik und Versorgung bei Eintritt und die Vorbereitung auf die Haftentlassung. Mehr klinisches und wissenschaftliches Engagement im Bereich Sucht und Haft ist dringend notwendig.

## Footnotes

1 https://www.bfarm.de/DE/Kodiersysteme/Klassifikationen/ICD/ICD-11/uebersetzung/_node.html. Letzter Zugriff 10.09.2025.

2 https://www.bundestag.de/resource/blob/915526/d5bd656ff388142b08b41be7057%20efe11/WD-9-056-22-pdf-data.pdf, letzter Zugriff 10.09.2025.

3 https://www.berlin.de/justizvollzug/service/zahlen-und-fakten/drogen-sucht/, letzter Zugriff am 17.10.2025.

4 https://www.awmf.org/service/awmf-aktuell/alkoholbezogene-stoerungen-screening-diagnose-und-behandlung, letzter Zugriff am 17.10.2025.

5 https://www.awmf.org/service/awmf-aktuell/medikamentenbezogene-stoerungen, letzter Zugriff am 17.10.2025.
